# Multi-Label Multi-Kernel Transfer Learning for Human Protein Subcellular Localization

**DOI:** 10.1371/journal.pone.0037716

**Published:** 2012-06-13

**Authors:** Suyu Mei

**Affiliations:** Software College, Shenyang Normal University, Shenyang, China; University of Jaén, Spain

## Abstract

Recent years have witnessed much progress in computational modelling for protein subcellular localization. However, the existing sequence-based predictive models demonstrate moderate or unsatisfactory performance, and the gene ontology (*GO*) based models may take the risk of performance overestimation for novel proteins. Furthermore, many human proteins have multiple subcellular locations, which renders the computational modelling more complicated. Up to the present, there are far few researches specialized for predicting the subcellular localization of human proteins that may reside in multiple cellular compartments. In this paper, we propose a multi-label multi-kernel transfer learning model for human protein subcellular localization (*MLMK-TLM*). *MLMK-TLM* proposes a multi-label confusion matrix, formally formulates three multi-labelling performance measures and adapts one-against-all multi-class probabilistic outputs to multi-label learning scenario, based on which to further extends our published work *GO*-*TLM* (gene ontology based transfer learning model for protein subcellular localization) and *MK-TLM* (multi-kernel transfer learning based on Chou's PseAAC formulation for protein submitochondria localization) for multiplex human protein subcellular localization. With the advantages of proper homolog knowledge transfer, comprehensive survey of model performance for novel protein and multi-labelling capability, *MLMK-TLM* will gain more practical applicability. The experiments on human protein benchmark dataset show that *MLMK-TLM* significantly outperforms the baseline model and demonstrates good multi-labelling ability for novel human proteins. Some findings (predictions) are validated by the latest *Swiss-Prot* database. The software can be freely downloaded at http://soft.synu.edu.cn/upload/msy.rar.

## Introduction

Recent years have witnessed much progress in computational modelling for protein subcellular localization [1]. However, researches on human genome and proteomics seem more urgent and important for human disease diagnosis and drug development. Unfortunately, there are far few specialized predictive models for human protein subcellular localization thus far [2], [3], [4], [5]. Furthermore, many human proteins have multiple subcellular locations, which renders the computational modelling more complicated. Up to the present, there are only two models (*Hum-mPLoc* [4] and *Hum-mPLoc 2.0* [5]) that can be applicable to multiple subcellular localization of human proteins.

Although many protein sequence feature extraction methods have been successfully developed for protein subcellular localization, such as *signal peptide* [6], *sequence domain* [7], *PSSM* [8], [9], *k*-mer [10], [11] etc., the accuracy of the models is still moderate or unsatisfactory, most of which average about 70% [6], [7], [9], [10], [11]. Garg A et al (2005) [3] used sequence features only (amino acid composition and its order information) for human protein subcellular localization, and the result is satisfactory (84.9%), but it covers only 4 subcellular locations. The Gene Ontology (*GO*) project has developed three structured controlled vocabularies (ontologies) that describe gene products in terms of their associated biological processes, cellular components and molecular functions in a species-independent manner, and the *GOA* database [12] provides high-quality electronic and manual associations (annotations) of *GO* terms to UniProt Knowledgebase (UniProtKB) entries [13]. Because the three aspects of gene ontology are closely related and the *GO* terms of cellular component contains direct indicative information about protein subcellular location, *GO* has become a generally effective feature for the prediction of protein subcellular localization [2], [4], [5], [14], [15], [16], [17], [18], [19], [20], [21], [22], [23], [24], [25], [26], [27], [28], [29]. Chou K.C. et al [2] proposed an ensemble leaning model called *Hum-PLoc* for human protein subcellular localization. The model consists of two parts: *GO*-based *kNN* and *PseAAC*-based *kNN*, and the latter part was designed to compensate for the model performance in the case of *GO* unavailability. To cover *multiplex* human proteins that reside in or transport across multiple subcellular locations, Shen HB et al [4] further proposed an improved model called *Hum-mPLoc*, which extended the number of subcellular locations from 12 to 14 and formally formulated the concept of *locative* protein and the success rate for multiplex protein subcellular localization. *Hum-PLoc*, *Hum-mPLoc* and the work [2], [15], [16], [17], [18], [19], [20], [21], [22] used the target protein's own *GO* information to train model, thus inapplicable to novel protein prediction. Many recent *GO*-based methods generally exploit the homolog *GO* information for novel protein subcellular localization [5], [23], [24], [25], [26], [27], [28], [29], [30]. Based on *Hum-mPLoc*, Shen HB et al [5] further proposed *Hum-mPLoc2.0* for multiplex and novel human protein subcellular localization, where a more stringent human dataset with 25% sequence similarity threshold is constructed to train a *kNN* ensemble classifier. *Hum-mPLoc2.0* incorporated those homologs with sequence similarity 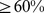, but achieved relatively low accuracy (62.7%). However, the method of setting threshold for homolog incorporation has the following disadvantages: (1) *significant* homolog (high sequence identity, assuming 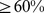) may potentially be *divergent* from the target protein in terms of protein subcellular localization, *for instance*, the target protein *P21291* resides in subcellular locations: *Nucleus*, while its significant homolog *P67966* (sequence identity: 90.16%; *PSI-Blast E*-value: 13e-174072, obtained by *Blast* default options) resides in subcellular locations: *Cytoplasm* and *Cytoskeleton*. High threshold of sequence identity, e.g. 60%, can not guarantee that no noise would be introduced to the target protein; (2) *remote* homolog (low sequence identity, assuming 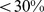) may be *convergent* to the target protein in terms of protein subcellular localization, *for instance*, the target protein *P21291* resides in subcellular locations: *Endoplasmic reticulum*, *Membrane* and *Microsome*, while its first 7 significant *remote* homologs queried against *SwissProt* 57.3 database [13] with default *Blast* option: *O75881(26.82%,4e-041),O02766(25.05%,4e-028), Q63688 (25.66%,2e-027),P22680(23.68%,4e-026),Q16850(23.92%,4e-025),O88962 (25.05%, 4e-025), Q64505 (23.13%, 1e-024)* (the first number in parenthesis denotes sequence identity and the second number denotes *PSI-Blast E*-value), also reside in the subcellular locations: *Endoplasmic reticulum*, *Membrane* and *Microsome*. High threshold of sequence identity (60%) would filter out all the *convergent* remote homologs that are informative to protein subcellular localization, and thus no homolog knowledge would be transferred to the target protein *P21291*. We can see that both *significant* homolog and *remote* homolog can be *convergent* homolog, or *divergent* homolog in terms of protein subcellular localization, thus we should conduct homolog knowledge transfer in a proper way, so that the noise from *divergent* homolog can be effectively depressed. Mei S et al [25] proposed a transfer learning model (gene ontology based transfer learning for protein subcellular localization, *GO-TLM*) to measure the individual contribution of *GO* three aspects to the model performance, where the kernel weights are evaluated by simple nonparametric cross validation. Mei S [26] further proposed an improved transfer learning model (*MK-TLM*), which conducted improvements on *GO-TLM* from the two major concerns: (1) more rational noise control over *divergent* homolog knowledge transfer; (2) comprehensive survey of model performance, especially for novel protein prediction. However, many human proteins reside in or transport across multiple cellular compartments, and the proteins with multiple locations may help reveal special biological implications to basic research and drug discovery [30], [31]. Neither *GO-TLM* nor *MK-TLM* is applicable to multiple protein subcellular localization prediction.

In this paper, we propose a multi-label multi-kernel transfer learning model for large-scale human protein subcellular localization (*MLMK-TLM*). Based on the work [25], [26], *MLMK-TLM* proposes a multi-label confusion matrix and adapts one-against-all multi-class probabilistic outputs to multi-label learning scenario. With the advantages of proper homolog knowledge transfer, comprehensive survey of model performance for novel protein and multi-labelling capability, *MLMK-TLM* gains more practical applicability. To validate *MLMK-TLM*'s effectiveness, we conduct a comprehensive model evaluation on the latest human protein dataset *Hum-mPLoc* 2.0 [5].

## Methods

### 1. Transfer learning

As a research field of machine learning community, transfer learning has attracted more and more attentions in recent years [32]. Traditional supervised learning generally assumes that all the data, including training data and unseen test data, are subjected to independent and identical distribution (*iid*), which doesn't hold true under many practical circumstances, especially in the field of biological data analysis. For example, the microarray gene expression data from different experimental platforms would be subjected to different level of experimental noise [33]. Transfer learning can be viewed as a bridge to transfer useful knowledge across two related domains with heterogeneous feature representations and different distributions. Pan S et al [32] reviewed the recent progress of transfer learning modelling and classified transfer learning into three categories based on the way of knowledge transfer: instance-based knowledge transfer [34], feature-based knowledge transfer [35] and parameter-based knowledge transfer [36].

Transfer learning modelling is generally conducted around three central dogmas: (1) how to define the relatedness between domains; (2) what to transfer; (3) how to transfer. In our work, we explicitly define the relatedness between protein sub-families and super-families by protein sequence evolution, i.e. protein homolog. Evolutionally closely-related proteins share similar subcellular localization patterns with high probability. Correspondingly, what to transfer is naturally the homolog *GO* term. Such a way of transfer learning modelling is computationally simple and biologically interpretable. In order to reduce the risk of *negative transfer*, *GO-TLM* [25] and *MK-TLM* [26] proposed a non-parametric multiple kernel learning method to measure the contribution of *GO* three aspects, target *GO* information and homolog *GO* information to the model performance. In this paper, we redefine confusion matrix, so that the *GO* kernel weights can be derived by cross validation for multi-label learning scenario.

### 2. *GO* feature construction

**Figure 1:**
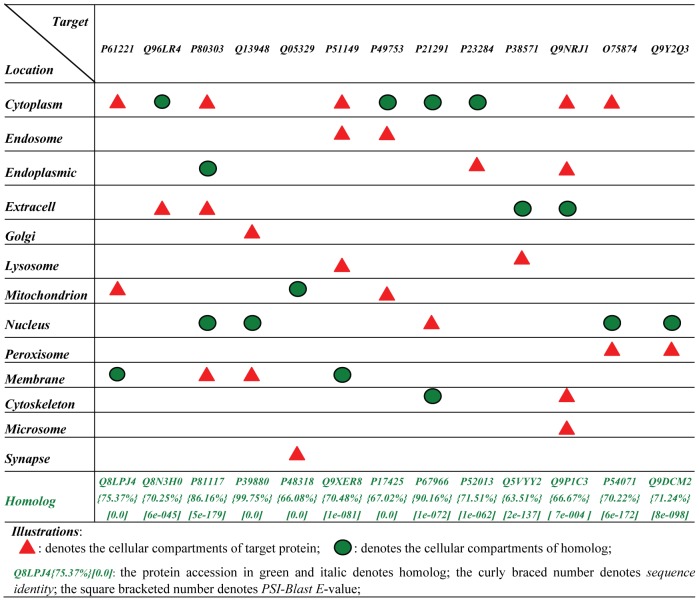
Illustration of *divergent* homolog in terms of subcellular localization.

All the proteins are represented with both the target *GO* terms and the homolog *GO* terms, which are extracted from *GOA* database [12] (77 Release, as of 30 November, 2009), and the homologs are extracted from *SwissProt* 57.3 database [13] using *PSI-Blast* [37]. Assume there are *u GO* terms *x_i_* (*i* = 1, 2,…, *u*), then protein 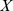 can be represented as follows:
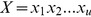
(1)If *GO* term *x_i_* is assigned to the protein *x* in *GOA* database, then *x_i_* = 1; Otherwise, *x_i_* = 0. To expressly estimate the individual contribution of the three *GO* aspects, *GO*-*TLM* [25] decomposed the feature vector (1) into the following three binary feature vectors:
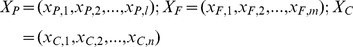
(2)However, *GO-TLM* aggregated the target *GO* information and the homolog *GO* information into one single feature vector, such that the two kinds of *GO* information are treated equally. We know that such a way of feature construction is not rational because *divergent* homolog *GO* information carries much noise. Figure 1 shows the difference of subcellular localization patterns between target human protein (*P61221* thru. *Q9Y2Q3*) and its homolog protein. The homologs are queried against *SwissProt* 57.3 database [13] with default *Blast* options (*E*-value: 10; substitution matrix: BLOSUM62). *E*-value is relaxed to 10 to obtain *remote* homologs for those proteins that have no *significant* homologs. For a target protein, we may encounter three cases for the selected homologs: (1) all homologs are *significant* homologs; (2) one part of homologs is *significant* homolog and the other part of homologs is *remote* homolog; (3) all homologs are *remote* homologs. Some *remote* homologs are *convergent* to the target protein in terms of protein subcellular localization (e.g. *remote* homologs *O75881*, *O02766*, *Q63688*, *P22680*, *Q16850*, *O88962* and *Q64505 to* target protein *P21291*), thus we should exploit the useful information from *remote* homologs; meanwhile, some *remote* homologs are *divergent* to the target protein, thus we should prevent *negative* knowledge transfer from the *remote* homolog. As compared to *remote* homolog, *significant* homolog is more likely to be *convergent* in terms of protein subcellular localization, but in some case, significant homolog is also likely to be *divergent*. Figure 1 lists one *divergent* homolog for each target protein. The illustrated *divergent* homolog has the highest sequence identity and *PSI-Blast E*-value among the target protein's divergent homologs. From Figure 1, we can see that the *significant* homologs reside in definitely distinct subcellular locations from the target protein, which implies that we should also depress noise from the *significant* homologs even though we encounter the above case (1). Similar to *MK-TLM* [26], we also separate the target *GO* information from its homolog *GO* information for the convenience of noise control. Here, we use *T* to denote the target protein and *H* to denote its homolog, thus the target *GO* feature vector is expressed as formula (3), and the homolog *GO* terms are aggregated into one homolog feature vector as formula (4):
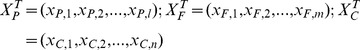
(3)

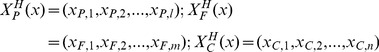
(4)Thus, each protein is represented by six binary feature vectors: 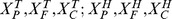.

### 3. Non-parametric multiple kernel learning

The six binary *GO* feature vectors {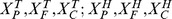} are used to derive six *GO* kernels {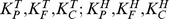}, and the *GO* kernels are further combined in the way that *MK-TLM* does [26]. In such a setting, higher homolog *GO* kernel weight implies more positive knowledge transfer, and lower homolog *GO* kernel weight can depress the potential noise by *divergent* homolog. Different to *MK-TLM*, *MLMK-TLM* adapts confusion matrix to multi-label learning scenario based on the concept of *locative* protein [4], [5]. For self-contained description and integrity, we give the full description of non-parametric kernel weight estimation in multi-label learning scenario as below, though some part of which is identical to *MK-TLM* [26]. Similar to *GO-TLM* and *MK-TLM*, the final kernel is defined as the following linear combination of sub-kernels:
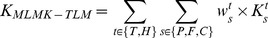
(5)

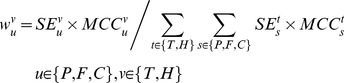
(6)Where *SE* denotes recall rate or sensitivity and *MCC* denotes Matthew's correlation coefficient. The kernel weights 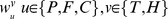 are derived by cross validation. Given a training dataset, we divide the training set into *k*-fold disjoint parts. For each fold cross validation, one part is used as validation set and the other parts are merged as training set to train the combined-kernel SVM. Thus, we can derive a confusion matrix *M* by evaluating the trained SVM against the test set. From the confusion matrix *M*, we can derive the kernel's *SE* and *MCC* measure as follows:
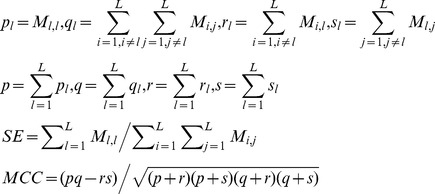
(7)Where, 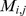 records the counts that class 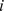 are classified to class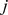; superscript 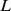 denotes subcellular locations; and all the other variables are intermediate variables that can be derived from the confusion matrix 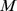.

In single-label learning scenario, 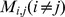 records the counts that class 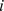 are misclassified to class 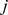, which is not applicable to multi-label learning scenario. Let's borrow the notion of *locative* protein [4], [5] to describe the multi-label confusion matrix. Assume that a protein *p* is located at two subcellular locations 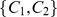, i.e., 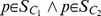 (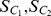 denote the set of proteins that reside in 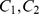, respectively), the notion of *locative* protein means that protein *p* can be viewed as two different proteins 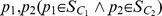. Now take 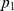 protein as test protein and the trained *SVM* labels 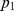 as follows:
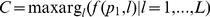
(8)Where, 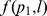 denotes the probability that protein 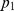 is assigned the label 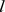 (see Section 4 of Methods for how to derive probability outputs). Thus, the *multi-label confusion matrix* can be defined as follows:
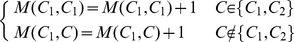
(9)Formula (9) shows that only if the predicted label of *locative* protein 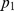 hits its true label 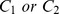, the prediction is deemed as *correct*; otherwise, the prediction would be deemed as *incorrect*.

As regards with kernel 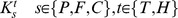, *Gaussian* kernel is used here:
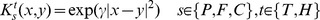
(10)


### 4. Multi-label learning

In our work, we extend *MK*-*TLM* [26] to multi-learning scenario based on one-against-all multi-class learning and binary *SVM* probability outputs [38]. Probability outputs tell us the confidence level that a query protein belong to each subcellular location, thus more intuitive and reasonable than ensemble voting [4], [5], [39] and label transfer of *kNN* nearest neighbour protein [27], [28], [30].

Assuming there are *K* subcellular locations, for each subcellular location *k*, we view the proteins that belong to *k* as positive examples and the proteins that belong to other subcellular locations except *k* as negative examples, based on which to train one binary *SVM*. Thus, we have *K* trained binary *SVM*s. If each binary *SVM* outputs {−1, +1} labels, multiple {+1} outputs can be viewed as multiple protein subcellular locations [40]. Because the {−1, +1} labels can not tell us the confidence level that a query protein belongs to each subcellular location, we don't adopt the method. If each binary *SVM* yields probability output, we can choose the label with the highest probability as the protein subcellular location, which is s-called one-against-all multi-class learning [38], [41]; if we set some probability threshold, the labels with probability over the threshold can be viewed as multiple protein subcellular locations, thus intuitively applicable to multi-label learning scenario. Platt J [41] proposed a method to adapt binary *SVM* {−1, +1} labels to posterior class probability as defined below:
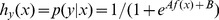
(11)Where the coefficient *A* and *B* can be derived from data by cross validation, and *f(x)* is uncalibrated decision value of binary *SVM*.

Actually, the one-against-all multi-class *SVM* with probability output has been implemented into the *LIBSVM* tool (http://www.csie.ntu.edu.tw/~cjlin/libsvm/), which can be easily used for multi-label learning. Only if we set *LIBSVM* prediction option “-b 1” (*LIBSVM* command option –b 1 means probability rather than {−1, +1} output), we can obtain the probability vector that a query protein is predicted to each subcellular location. By setting optimal probability threshold, we can determine the optimal multiple labelling for the query protein based on the predicted probability vector.

### 5. Model evaluation and model selection

The existing *GO*-based models only reported the *optimistic* performance by evaluating the proposed model against information-rich (*GO*, *PPI*, *image*) test proteins, and seldom reported the performance for novel proteins [4], [5], [14], [15], [16], [17], [18], [19], [20], [21], [22], [23], [24], [25], [27], [28], [29], [30]. Apparently, the *optimistic* performance is not enough to be a comprehensive survey of the model's true predictive ability, especially for novel protein prediction. *MK-TLM* [26] attempted to conduct a comprehensive survey of the model performance in *optimistic*, *moderate* and *pessimistic* cases, and demonstrated good performance for novel proteins and those proteins that belong to the protein family we know little about. In this paper, the proposed *MLMK-TLM* inherits all *MK-TLM*'s advantages. The *Optimistic* case means the training set and the test set both abound in *GO* information; the *Moderate* case means that the test set contains no *GO* information at all, which can be simulated by removing the test kernels 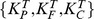; the *Pessimistic* case means that both the training set and the test set contains no *GO* information at all where the target *GO* information is removed from both the training set and the test set, which can be simulated by removing the training kernels 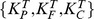 and test kernels 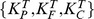.

The performance evaluation under multi-label learning scenario seems more complicated as compared to single-label learning scenario. Because the model performance estimation involves both *singlex* protein (only one subcellular location) and *multiplex* protein (multiple subcellular locations), we should conduct two performance estimation experiments: one experiment is *overall* performance estimation on *locative* dataset, where *multiplex* protein is viewed as multiple *singlex* proteins as *Hum-mPLoc* 2.0 [4], *Virus-mPLoc* [15], *iLoc-Euk* [27], *iLoc-Virus* [28] and *Plant-mPLoc* [30] did; the other experiment is multi-labelling estimation for *multiplex* proteins. The first experiment is similar to traditional supervised learning estimation except that multi-label confusion matrix is adopted instead (see formula 8 & 9); in the second experiment, cross validation is conducted on *multiplex* proteins only and the *singlex* proteins are always treated as training data. Thus, the whole training set is composed of two parts: *fixed part* from the *singlex* proteins and the *variable part* from the *multiplex* proteins. In addition, the model performance estimation in the second experiment is much more complicated. To simplify the formulation, lets' first give several symbol annotations: (1) 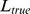 denotes the true label set of a *multiplex* protein *p*; (2) 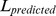 denotes the predicted label set of a *multiplex* protein *p*; (3) 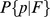 denotes the protein set *P* whose protein *p* satisfies the condition *F*; (4)  denotes set cardinal; (5) minus symbol  denotes set difference; (6) 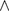 denotes *logic AND*. Based on the symbols, we can formally define *Label Hit Rate* (*LHR*), *Perfect Label Match Rate* (*PLMR*) and *Non-target Label Hit Rate* (*NT-LHR*) as follows:
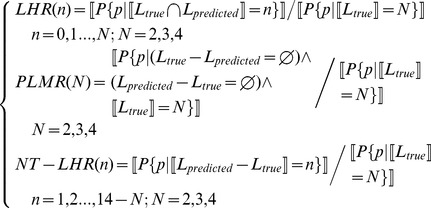
(12)where *N* denotes the number of subcellular locations a protein may reside in, with maximum value *4* here; *n* denotes the number of correct *HIT*s or wrong *HIT*S, with maximum value 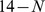 here (we assume the total number of subcellular locations is *14*). The *multiplex* proteins in *Hum-mPLoc 2.0* [5] can be divided into 3 subsets that possesses *2*, *3* and *4* labels (subcellular locations), respectively. We will report *LHR*, *PLMR* and *NT-LHR* on each subset. Take *2-label* subset as example, the prediction may hit *0*, *1* or *2* true labels. Low *0*-label hit rate and high *1*- and/or *2*-label hit rate imply good model performance. However, the prediction may also hit 1∼12 non-target labels (excluding *2* true labels from total *14* subcellular locations). High *NT-LHR* implies high misleading tendency, which should be as low as possible. The existing multi-label learning model for protein subcellular localization [4], [5], [15], [19], [27], [28], [29], [30] seldom reported *NT-LHR*. If the prediction hits the true labels and yields no other misleading labels, we call the case *perfect label match*; otherwise, we call the case *non-perfect label match*. High *Perfect Label Match Rate* (*PLMR*) implies good predictive ability and low misleading tendency.

*MLMK-TLM* is a relatively complex model that requires time-consuming computation for model comparison and model selection. Apart from *SVM* regularization parameter *C* and kernel parameter 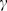, *MLMK-TLM* introduces a hyper-parameter 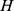 that denotes the number of homologs for knowledge transfer. Assume there are *N* proteins in the dataset and the hyper-parameter sets are 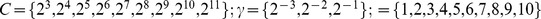, *MLMK-TLM* has to fix one hyper-parameter to optimize the other hyper-parameters, and in each iteration has to compute kernel matrices, thus the computational complexity is 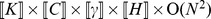, where 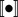 denotes set cardinal, 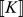 denotes the number of kernel matrices and O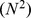 denotes the computational complexity for kernel computing. For large-scale human protein dataset *Hum-mPLoc* 2.0 [5], the model selection is rather time-consuming. Hence, we adopt 5-fold cross validation instead of leave-one-out cross validation (*LOOCV*) (*Jackknife*) as *GO-TLM* [25] and *MK-TLM* [26] did. For multi-labelling estimation, the *multiplex* proteins are divided into 5 nearly-even parts, one part as test set, and the other parts are merged with the *singlex* proteins into training set, thus iterates for 5 times until all the *multiplex* proteins participate in the performance estimation process (see Section 6 of Results).

For performance estimation on *locative* proteins, we adopt the performance measures: Sensitivity (*SE*), Specificity (*SP*), Matthew's correlation coefficient (*MCC*), *Overall MCC*, and *Overall Accuracy*. For multi-labelling estimation, we adopt *LHR*, *PLMR* and *NT-LHR*.

## Results

### 1. Dataset

Shen HB et al [5] constructed a large-scale human protein dataset. The dataset covers 14 subcellular locations and contains 3106 distinct human proteins, where 2580 proteins belong to one subcellular location, 480 to two locations, 43 to three locations, and 3 to four locations. The protein with multiple subcellular locations should be treated as one training example of each subcellular location it belongs to, thus the same protein should be viewed as different protein within different subcellular location, referred to as *locative* protein in the literatures [4], [5], [15], [19], [27], [28], [29], [30]. Thus, there are 3681 *locative* proteins in the dataset [5]. The dataset is a good benchmark for model performance comparison, because none of the proteins has ≥25% sequence identity to any other proteins in the same subcellular location. Accordingly, we choose *Hum-mPLoc* 2.0 [5] as the baseline models for performance comparison. Although the dataset [40] collected much more *multiplex* human proteins, we don't use it to evaluate the multi-labelling, because the sequence similarity reaches 80%, so high as to yield performance overestimation.

### 2. Model performance evaluation

#### 2.1 *Optimistic* case: both training set and test set abound in target *GO* information

The *optimistic* case assumes that both the training set and the test set abound in target *GO* information, that's, the training proteins and the test protein by themselves contain rich *GO* information before incorporating the homolog *GO* information. We call this case *MLMK-TLM-I*. As shown in *MLMK-TLM-I* section of Table 1, *MLMK-TLM* achieves 87.04% accuracy and 0.8606 *MCC* on *Hum-mPLoc* 2.0 human protein data, significantly outperforming the baseline *Hum-mPLoc* 2.0 62.7% [5]. Actually, *Hum-mPLoc* 2.0 aggregated the target protein's *GO* information together with the homolog *GO* information to train classifier, thus the overall accuracy 62.7% is the model's *optimistic* performance. The optimal hyper-parameter setting is 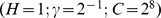, where 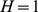 means that only one homolog *GO* information is transferred to the target protein. The high *MCC* value (0.8606) implies that *MLMK-TLM* achieves good predictive balance among the 14 human protein subcellular locations. We can see from *MLMK-TLM-I* section of Table 1 that *MLMK-TLM* achieves good model performance one most subcellular locations, even the small *Peroxisome* (*SP* = 0.9750; *SE* = 0.8298; *MCC* = 0.8983) and *Microsome* (*SP* = 0.9500; *SE* = 0.7917; *MCC* = 0.8665). *MLMK-TLM* relatively underperforms on the small subcellular locations: *Endosome* (*SP* = 0.9167; *SE = 0.4583*; *MCC = 0.6467*) and *Synapse* (*SP = 1.0000*; *SE = 0.5455*; *MCC = 0.7375*). The poor performance may result from the sources: (1) small number of training proteins; (2) the target *GO* information is not as rich as the other subcellular locations; (3) the homolog *GO* information may be more divergent.

**Table 1:** Optimal performance on *3681* human *locative* protein dataset.

*Subcellular location*	*Size*	*MLMK-TLM-I (optimistic)* 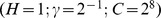			*MLMK-TLM-II (moderate)* 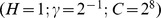			*MLMK-TLM-III (pessimistic)* 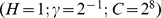		
		*SP*	*SE*	*MCC*	*SP*	*SE*	*MCC*	*SP*	*SE*	*MCC*
***Centrosome***	77	0.9063	0.7532	0.8229	0.8772	0.6494	0.7504	0.8235	0.7273	0.7694
***Cytoplasm***	817	0.7845	0.8556	0.7704	0.8061	0.8042	0.7552	0.7380	0.8446	0.7322
***Cytoskeleton***	79	0.9123	0.6582	0.7710	0.7910	0.6709	0.7231	0.8333	0.6329	0.7212
***Endosome***	24	0.9167	0.4583	0.6467	0.8000	0.5000	0.6306	0.9167	0.4583	0.6467
***Endoplasmic reticulum***	229	0.9302	0.8734	0.8951	0.9151	0.8472	0.8730	0.8818	0.8472	0.8557
***Extracell***	385	0.9525	0.8857	0.9096	0.9284	0.8753	0.8906	0.9413	0.8753	0.8977
***Golgi apparatus***	161	0.9214	0.8012	0.8534	0.9161	0.8137	0.8576	0.8705	0.7516	0.8010
***Lysosome***	77	1.0000	0.7143	0.8426	0.9828	0.7403	0.8504	0.9825	0.7273	0.8426
***Microsome***	24	0.9500	0.7917	0.8665	0.8947	0.7083	0.7949	0.8947	0.7083	0.7949
***Mitochondrion***	364	0.9620	0.9038	0.9255	0.9477	0.8956	0.9131	0.9339	0.8544	0.8825
***Nucleus***	1021	0.8479	0.9334	0.8486	0.8156	0.9314	0.8238	0.8287	0.8815	0.8028
***Peroxisome***	47	0.9750	0.8298	0.8983	0.9318	0.8723	0.9004	0.9762	0.8723	0.9219
***Plasma membrane***	354	0.8746	0.8672	0.8576	0.8169	0.8446	0.8130	0.8680	0.8362	0.8370
***Synapse***	22	1.0000	0.5455	0.7375	1.0000	0.5455	0.7375	1.0000	0.5000	0.7060
***Overall Accuracy/MCC***		87.04%/0.8606			85.22%/0.8411			83.97%/0.8277		

#### 2.2 *Moderate* case: training set abounds in target *GO* information while test set contains no target *GO* information

The most common scenario we encounter may be that we have a plenty of well-annotated training proteins and need to label some novel proteins at hand. We call the scenario as *moderate* case, referred to as *MLMK-TLM-II*. Novel proteins generally have no *GO* information at all. Most of the existing *GO*-based models except the work [26] ignored performance estimation in this case. Once the proposed models work in such a scenario, the performance may not be as *optimistic* as reported. Therefore, experiments should be expressly designed for the *moderate* case to test *MLMK-TLM*'s applicability to novel proteins.

The test procedure for *moderate* case seems more complicated than that for *optimistic* case, because the proteins in the test set have no target *GO* information. Thus, the three target test kernels 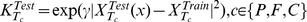 can not be derived, because 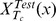 is null (superscript *Test* denotes test set and *Train* denotes training set). For the reason, we substitute the homolog *GO* feature vector of test protein for its target *GO* feature vector to calculate the test kernel as follows:
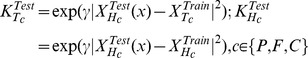
(13)


As shown in *MLMK-TLM-II* section of Table 1, *MLMK-TLM* achieves 85.22% accuracy and 0.8411 *MCC* on the benchmark data, still significantly outperforming the baseline *Hum-mPLoc* 2.0 62.7% [5] and nearly 2% lower than the *optimistic* case (87.04% accuracy; 0.8606 MCC). Except for small performance decrease, *MLMK-TLM-II* demonstrates similar behaviour to *MLMK-TLM-I* (predictive balance and relative poor performance on *Endosome* & *Synapse*, see the underscored *SE* measures). The results show that *MLMK-TLM* is convincingly applicable to novel protein prediction, and the performance decrease shows that it is necessary to expressly report the model performance in *moderate* case.

#### 2.3 *Pessimistic* case: both training set and test set contain no target *GO* information

In this section, we study an extreme case, called *pessimistic* case, where a protein subfamily or species is not *GO*-annotated at all, that's, we know nothing about the protein subfamily or species but the protein sequence information. The key point is whether the homolog *GO* information is informative enough to train an effective prediction model for the protein subfamily or species we know little about. To validate the point, we assume that at least one *GO*-annotated homolog can be queried for the target protein, which is not restrictive with the rapid progress of *GOA* database [12]. If experimental results support the idea, *MLMK*-*TLM* will gain much wider application. Different from the *optimistic* case and the *moderate* case, the *pessimistic* test procedure contains only three homolog *GO* kernels with target *GO* kernels missing.

As shown in *MLMK-TLM-III* section of Table 1, *MLMK-TLM* achieves 83.97% accuracy and 0.8277 *MCC* on the benchmark data, significantly outperforming the baseline *Hum-mPLoc* 2.0 62.7% [5], nearly 3% lower than the *optimistic* case (87.04% accuracy; 0.8606 MCC) and nearly 1.5% lower than the *moderate* case (85.22% accuracy; 0.8411 *MCC*). Similarly, *MLMK-TLM-II* demonstrates similar behaviour to *MLMK-TLM-I* & *MLMK-TLM-I* (predictive balance and relative poor performance on *Endosome* & *Synapse*, see the underscored *SE* measures). The results show that *MLMK-TLM* is applicable to novel protein prediction for the protein subfamily or species that we know little about.

### 3. Optimal number of homologs

*Homolog* is a good bridge for knowledge transfer between two evolutionarily- related protein subfamilies, super-families or species. However, biological evidences demonstrate that *divergent* homologs are subjected to different subcellular localization patterns from the target protein (see Figure 1), thus incorporating *divergent* homologs would leads to *negative transfer* and do harm to model performance. Thus, it is highly required to quantitatively study how much homolog *GO* information should be transferred to the target protein. Most of the existing *Homolog*-*GO*-based models except the work [26] seldom conducted the quantitative analysis. Because the homolog space is generally quite huge, the model selection is unendurably long if the hyper-parameter *H* is large, so we empirically define the homolog search space as 7 homologs with the most significant *E*-value.

**Figure 2:**
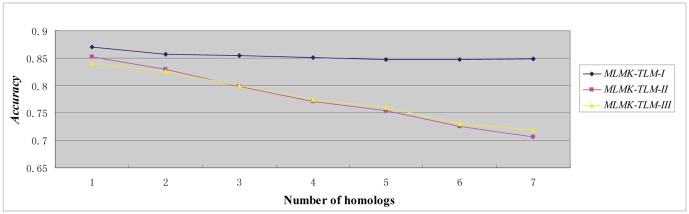
Performance on *3681* human protein dataset with varying homologs.

As shown in Figure 2, the optimal number of homologs is 1 for *optimistic* case (*MLMK-TLM-I*), *moderate* case (*MLMK-TLM-II*), and *pessimistic* case (*MLMK-TLM-III*). The model performance slightly decreases for the *optimistic* case (*MLMK-TLM-I*) with the incorporation of more homologs, while the model performance decreases sharply for the *moderate* (*MLMK-TLM-II*) & *pessimistic* case (*MLMK-TLM-III*). When the number of homologs reaches 7, the accuracy sharply drops about 15% for *moderate* & *pessimistic* case. We can see that *divergent* homologs adversely contribute little to the *optimistic* case, partly because the target protein's own *GO* information can counteract the unfavourable impact of the *divergent* homolog *GO* information. For the *moderate* & *pessimistic* case, the unfavourable *divergent* homolog *GO* information greatly deteriorates the model performance. From the results, we can safely conclude that it is highly necessary to quantitatively study how much homolog *GO* information should be transferred to the target protein.

It is worthy noting that the *pessimistic* case contains no target *GO* information but slightly outperforms the *moderate* case beyond our expectation (except at the first & second points of the curve in Figure 2). The reason may be that the substitution of the homolog *GO* feature vector for the target *GO* feature vector results in the slight performance deterioration (see *Formula 13*).

### 4. Kernel weight distribution

The *GO* kernel weights are evaluated using *3*-fold cross validation as described in Section 3 of Methods, rather than 5-fold cross validation as *GO-TLM* [25] conducted, because the additional hyper-parameter *H* makes the model selection more time-consuming. Actually, to evaluate the model performance, we conduct two-level cross validation: the outer *5*-fold cross validation uses the whole dataset to evaluate performance, and the inner *3*-fold cross validation uses the training set from the outer cross validation to estimate the kernel weights. Similar to *GO-TLM* [25] and *MK-TLM* [26], the kernel weight distributions yielded from the outer *5*-fold cross validation is quite similar, so we choose one typical kernel weight distribution to illustrate the *GO* kernels' contribution to the model performance.

**Figure 3:**
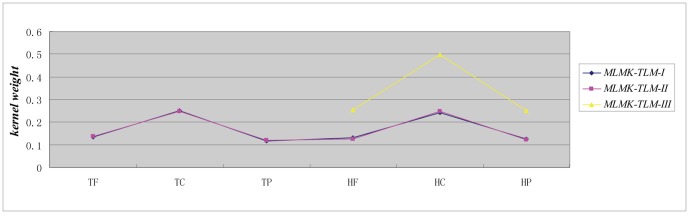
Kernel weight estimation on *3681* human *locative* protein dataset.

As shown in Figure 3, the *x* axis denotes the six *GO* kernels, where *T* denotes target, *H* denotes homolog, *F*, *C* and *P* denote the three aspects of gene ontology (molecular function, cellular compartment and biological process), respectively. We can see that both the *optimistic* case and the *moderate* case have similar kernel weight distributions on the benchmark dataset, while the *pessimistic* case is similar to the homolog *GO* kernel weight distribution of the *optimistic* case and the *moderate* case (see the latter part of curve in Figure 3) (the *pessimistic* case contains only three homology *GO* kernels in that the target protein's *GO* information is missing). No matter the target *GO* kernels or the homolog *GO* kernels, *C* (cellular component) demonstrates much higher kernel weight. For *optimistic* case and *moderate* case, both the target *GO* kernels and the homolog *GO* kernels make equivalent contribution to the model performance (compare the former half part and the latter half part of the curve in Figure 3). From the results, we can conclude that the homolog knowledge transfer is instrumental to novel target protein research.

### 5. Multi-labelling estimation

As stated in Section 4 of Methods, *MLMK-TLM* can yield the probability outputs from *Formula 11*. We can assign to the test protein the subcellular locations whose predicted probability is greater than the optimal probability threshold. The threshold setting should achieve rational balance between higher *LHR* (*Label Hit Rate*) & *PLMR* (*Perfect Label Match Rate*) and lower *NT-LHR* (*Non-target Label Hit Rate*) defined by *Formula 12* in Section 5 of Methods. Generally, higher *LHR* & *PLMR* also implies higher *NT-LHR*. In the work, the optimal probability threshold is selected from 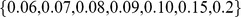. Besides *LHR*, *PLMR* and *NT-LHR* (Table 2 thru. Table 4), we also list some proteins of *perfect label match* (Table 5) and *non-perfect label match* (Table 6) to demonstrate *MLMK-TLM*'s multi-labelling ability. Interestingly, some *non-target label hits* in terms of the original *Hum-mPLoc* 2.0 dataset (*GOA database version 70.0 released March 10 2008*) are validated as *TRUE LABEL* (*Correct Prediction*) by the latest *Swiss-Prot* database (*UniProt release 2011_11 Nov 16, 2011*, http://www.uniprot.org/) (see Table 6).

**Table 2:** Multi-labelling evaluation for *optimistic* case.

*Multiplex Locations*	*Size*	*Label Hit Rate*					*PLMR*	*Non-target Label Hit Rate*			
		*0*	*1*	*2*	*3*	*4*		*1*	*2*	*3*	*4*
***2***	480	3.33%	*38.12%*	**58.54%**	0	0	42.92%	18.96%	8.54%	2.92%	0.42%
***3***	43	2.33%	*25.58%*	*44.19%*	**27.91%**	0	13.95%	25.58%	16.28%	2.33%	0
***4***	3	0	*33.33%*	*0*	*33.33%*	**33.33%**	33.33%	0	0	33.33%	0

**Table 3:** Multi-labelling evaluation for *moderate* case.

*Multiplex Locations*	*Size*	*Label Hit Rate*					*PLMR*	*Non-target Label Hit Rate*			
		*0*	*1*	*2*	*3*	*4*		*1*	*2*	*3*	*4*
***2***	480	4.37%	*38.75%*	**56.87%**	0	0	38.75%	22.29%	9.38%	3.96%	0.63%
***3***	43	0	*25.58%*	*48.84%*	**25.58%**	0	9.30%	20.93%	25.58%	2.33%	0
***4***	3	0	*33.33%*	*33.33%*	*33.33%*	**0**	0	33.33%	33.33%	0	0

**Table 4:** Multi-labelling evaluation for *pessimistic* case.

*Multiplex Locations*	*Size*	*Label Hit Rate*					*PLMR*	*Non-target Label Hit Rate*			
		*0*	*1*	*2*	*3*	*4*		*1*	*2*	*3*	*4*
***2***	480	3.75%	*38.12%*	**58.13%**	0	0	34.58%	24.58%	11.67%	4.79%	2.50%
***3***	43	0	*25.58%*	*41.86%*	**32.56%**	0	9.30%	25.58%	18.60%	11.63%	0
***4***	3	0	*33.33%*	*0*	66.67%	**33.33%**	0	0	33.33%	0	33.33%

**Table 5:** Multi-labelling evaluation—*perfect label match.*

	*Protein Accessions*
***Optimistic case*** ***[> = 0.09]***	Q9UHB9;Q9UBQ5;Q9BT78;Q6SJ96;Q86W56;Q14145;Q9GZM5;Q9ULJ6;Q96Q15;Q15025;P06734;Q9NTJ3;O95391;Q92973;Q9UNS2;Q53HL2;Q9BZZ5;O43592;Q13352;Q9UHD9;Q9UHB6;Q9UQ80;P61221;Q9C0E2;P61011;P35520;O43324;Q9UI26;Q14738;P04792;Q9NYL5;Q7L7V1;Q86VP3;Q15392;Q7Z699;Q07954;Q13421;P11532;Q15004;Q12794;Q9P1T7;Q96QU8;Q9GZU1;Q9UKA2;O95070;P00488;O95153;Q12830;P16989;Q9Y6A2;Q92934;O60869;Q8N2I9;Q8N488;P38936;P16442;Q14493;Q9Y282;Q9HAP2;Q92551;Q9Y3C4;Q9NS86;Q96DU7;Q14978;P07954;Q9NXE4;P37840;Q9NRA8;P15907;Q08211;O76054;Q02880;O95997;Q8TEM1;P24071;Q969M3;Q9Y251;O95406;Q8NFM7;Q8WXI7;P14060;Q92820;P30519;Q96S99;Q7Z417;O95163;P23490;Q9BUP3;Q96Q89;Q96FX2;Q9Y6K9;O43157;O75533;Q49AN0;O75312;P56693;O60921;O95456;Q9Y6Q9;Q9BZG8;Q8N668;Q86X55;P35052;Q86UB2;Q99436;P29728;Q96PL5;Q9NY26;O14492;O15360;O75419;Q15020;P31512;O95273;Q8TEQ6;Q96PM5;Q9UNY4;O14980;Q9BV57;Q92681;Q6AZY7;O95479;Q9NZ42;O43174;Q93084;P20309;Q71F23;Q08J23;Q99519;Q86UW9;Q49MI3;P52630;P62826;Q00597;P61457;O00628;P60900;Q9H8E8;Q96C86;Q9BZJ0;Q9H8T0;Q9UKT4;Q12934;Q06787;Q13485;Q8IVL5;Q6RW13;Q8IWL8;Q13363;Q9Y314;P55060;Q9GZY1;O95453;Q07955;Q8WXG1;Q9C000;Q6IMN6;Q13765;O94829;Q9BRS8;O75365;P13987;O15354;Q9NSB8;Q14512;Q8TC92;Q96BI3;Q9NPH3;Q14703;P78329;Q04656;P58340;Q15276;O75884;Q7L5N1;Q969Q6;Q96KS0;Q86UA6;Q9NQW1;Q13098;Q7Z4G1;Q9BWU0;Q9BWS9;P21580;Q9UN88;Q13868;Q9NPD3;O14929;O15304;P61960;Q15650;Q13231;Q9NXR7;Q86YF9;P08684;Q9NWZ8;Q9BPZ7;P50748;Q03933;Q9NYF8;Q9Y397;Q96FF9;Q14534;P50542;Q00978;Q9H9T3;Q11203;O75881;Q9Y6K0;Q9P212;P43681;O43493;Q9UM00;
***Moderate case*** ***[> = 0.08]***	Q9UHB9;Q9UBQ5;Q9BT78;Q6SJ96;Q86W56;Q9GZM5;Q9ULJ6;O43663;Q96Q15;Q15025;Q9NTJ3;O95391;Q92973;Q9UNS2;Q53HL2;Q8IZY2;Q9BZZ5;O43592;Q13352;Q9UHB6;Q9UQ80;Q9C0E2;P22059;P61011;P35520;O43324;Q9UI26;Q14738;P04792;Q9NYL5;Q7L7V1;Q86VP3;Q15392;Q9UHK6;Q7Z699;P08962;Q15004;Q12794;Q9P1T7;Q96QU8;Q9GZU1;Q9UKA2;O95070;P00488;O95153;Q12830;P16989;Q13316;Q9Y6A2;Q92934;O60869;Q8N2I9;Q8N488;P38936;P16442;Q14493;Q9Y282;Q9HAP2;Q92551;Q9Y3C4;Q9NS86;Q96DU7;Q14978;P07954;Q9NXE4;Q9Y5Z9;P37840;Q9NRA8;P15907;Q08211;O76054;Q02880;O95997;Q8TEM1;Q969M3;Q9Y251;O95406;Q8WXI7;P14060;Q92820;P30519;Q96S99;Q7Z417;O95163;Q9BUP3;Q96Q89;Q96FX2;Q9Y6K9;O75533;Q49AN0;O75312;P56693;O60921;O95456;Q9Y6Q9;Q9BZG8;Q8N668;Q86X55;P35052;Q86UB2;Q99436;Q96PL5;O15360;O75419;Q15020;O95273;Q8TEQ6;Q96PM5;Q9UNY4;O14980;Q9BV57;Q92681;O95479;Q9NZ42;O43174;Q8ND25;P20309;Q71F23;Q08J23;Q99519;Q86UW9;P52630;P62826;Q00597;P61457;O00628;Q9H8E8;Q96C86;Q9BZJ0;Q9UKT4;Q12934;Q06787;Q13485;Q6RW13;Q8IWL8;Q13363;Q9Y314;P55060;Q9GZY1;O95453;Q07955;Q9C000;Q13765;O94829;Q9BRS8;O15354;Q9NSB8;Q14512;Q8TC92;Q96BI3;Q9NPH3;Q14703;P78329;P58340;Q15276;O75884;Q7L5N1;Q969Q6;Q96KS0;Q86UA6;Q92624;Q13098;Q7Z4G1;Q9BWU0;Q9BWS9;P21580;Q13868;Q9NPD3;O14929;P61960;Q15650;Q13231;Q9NXR7;Q86YF9;P08684;Q03135;Q9NWZ8;Q9BPZ7;P50748;Q03933;Q9NYF8;Q9Y397;Q96FF9;Q14534;P50542;Q00978;Q11203;O75881;Q9Y6K0;Q9P212;
***Pessimistic case*** ***[> = 0.07]***	Q9UBQ5;Q9BT78;Q6SJ96;Q86W56;Q9ULJ6;O43663;Q96Q15;Q15025;P06734;Q9NTJ3;O95391;Q92973;Q9UNS2;Q53HL2;Q9BZZ5;O43592;Q13352;Q9UHB6;Q9UQ80;Q9C0E2;P61011;P35520;O43324;Q9UI26;Q14738;P04792;Q9NYL5;Q7L7V1;Q15392;Q9UHK6;Q7Z699;P11532;Q15004;Q12794;Q9P1T7;Q96QU8;Q9UKA2;A5X5Y0;P00488;O95153;Q12830;P16989;Q9Y6A2;Q92934;O60869;Q8N488;P38936;P16442;Q14493;Q9HAP2;Q92551;Q9Y3C4;Q9NS86;Q96DU7;Q14978;P07954;Q9NXE4;Q9Y5Z9;P37840;Q9NRA8;P15907;Q08211;O76054;Q02880;O95997;Q9UKL3;P24071;Q969M3;Q9Y251;O95406;Q92820;P30519;Q96S99;Q7Z417;O95163;Q9BUP3;Q96Q89;Q96FX2;O75533;Q49AN0;P56693;O60921;O95456;Q9Y6Q9;Q9BZG8;Q86X55;P35052;P58335;Q86UB2;Q99436;Q96PL5;O15360;O75419;Q15020;O95273;Q8TEQ6;Q96PM5;Q9UNY4;O14980;Q9BV57;Q92681;Q9NZ42;O43174;P20309;Q71F23;Q08J23;Q86UW9;P52630;P62826;Q00597;P61457;Q9H8E8;Q96C86;Q9BZJ0;Q08378;Q9UKT4;Q06787;Q13485;Q8IWL8;Q13363;Q9Y314;P55060;O95453;Q07955;Q9C000;Q6IMN6;Q13765;O94829;Q9BRS8;Q9UKT7;P49721;Q14512;Q8TC92;Q96BI3;Q9NPH3;Q14703;P8329;Q04656;P58340;O75884;Q7L5N1;Q969Q6;Q96KS0;Q86UA6;Q92624;Q13439;Q13098;Q7Z4G1;Q9BWU0;Q9BWS9;P21580;Q13868;Q9NPD3;O14929;P61960;Q13231;Q9NXR7;P08684;Q9NWZ8;P50748;Q03933;Q9NYF8;Q9Y397;Q96FF9;Q14534;Q00978;Q53QV2;Q9H9T3;Q11203;O75881;

**Table 6:** Multi-labelling evaluation—*non*-*perfect label match*.

		*True Subcellular Locations*	*Predicted Subcellular Locations*
***Optimistic Case*** ***[> = 0.09]***	***O43663***	***Cytoplasm; Nucleus***	***Nucleus[0.471]; Cytoplasm[0.365]; Cytoskeleton[0.136]***
	***Q92797***	***Cytoskeleton; Nucleus***	***Nucleus[0.470]; Cytoplasm[0.257]; Plasma membrane[0.129]***
	***Q92597***	***Cytoplasm; Nucleus; Plasma membrane***	***Nucleus[0.588]; Cytoplasm[0.333]***
	***P30533***	***Cytoplasm; Endoplasmic reticulum***	***Endoplasmic reticulum[0.237]; Cytoplasm[0.229]*** ***Nucleus[0.115]; Plasma membrane[0.181]***
	***Q9HD36***	***Mitochondrion; Nucleus***	***Nucleus[0.562];Mitochondrion[0.160];Cytoplasm[0.156]***
	***P22059***	***Cytoplasm; Golgi apparatus***	***Golgi apparatus[0.837]***
	***Q86YR5***	***Endoplasmic reticulum; Golgi apparatus***	***Golgi apparatus[0.454]; Endoplasmic reticulum[0.316]; Cytoplasm[0.091]***
	***P41222***	***Endoplasmic reticulum; Golgi apparatus; Nucleus***	***Endoplasmic reticulum[0.237]; Nucleus[0.207]*** ***Extracell[0.146]; Cytoplasm[0.143]; Golgi apparatus[0.107];***
***Moderate Case*** ***[> = 0.08]***	***P15941***	***Cytoplasm; Nucleus***	***Plasma membrane[0.467]; Nucleus[0.178]; Cytoplasm[0.145]***
	***Q14145***	***Cytoplasm; Nucleus***	***Cytoplasm[0.520]; Nucleus[0.292]; Endoplasmic reticulum[0.089]***
	***Q86YR5***	***Endoplasmic reticulum; Golgi apparatus***	***Golgi apparatus[0.371]; Endoplasmic reticulum[0.272]; Cytoplasm[0.129];Plasma membrane[0.124]***
	***P41222***	***Endoplasmic reticulum;Golgi apparatus*** ***Nucleus***	***Nucleus[0.257];Endoplasmic reticulum[0.214]; Cytoplasm[0.166]; Extracell[0.103]; Golgi apparatus[0.098]***
	***Q96JC1***	***Cytoplasm; Lysosome***	***Lysosome[0.676]; Endosome[0.208]***
	***Q96EY5***	***Cytoplasm; Nucleus***	***Nucleus[0.223]; Endosome[0.202]; Golgi apparatus[0.121]*** ***Cytoskeleton[0.092]***
	***Q86WA9***	***Endoplasmic reticulum;Golgi apparatus*** ***Plasma membrane***	***Lysosome[0.642]; Endoplasmic reticulum[0.136]*** ***Plasma membrane[0.101]***
	***Q9NWZ5***	***Cytoplasm; Nucleus***	***Endoplasmic reticulum[0.459]; nucleus[0.320]; Cytoplasm[0.133]***
***Pessimistic Case [> = 0.07]***	***Q9UHD9***	***Cytoplasm; Nucleus***	***Endoplasmic reticulum[0.315]; Nucleus[0.284]; Cytoplasm[0.202]***
	***P30533***	***Cytoplasm; Endoplasmic reticulum***	***Endoplasmic reticulum[0.250]; Cytoplasm[0.237]*** ***Nucleus[0.151]; Plasma membrane[0.139]***
	***Q86YR5***	***Endoplasmic reticulum;Golgi apparatus***	***Golgi apparatus[0.421]; Endoplasmic reticulum[0.202]*** ***Cytoplasm[0.136]; Plasma membrane[0.106]***
	***Q9NX74***	***Cytoplasm; Endoplasmic reticulum***	***Endoplasmic reticulum[0.273]; Cytoplasm[0.271]*** ***Mitochondrion[0.247]***
	***P41222***	***Endoplasmic reticulum;Golgi apparatus*** ***Nucleus***	***Nucleus[0.224]; Endoplasmic reticulum[0.197]; Cytoplasm[0.184]; Extracell[0.126];Golgi apparatus[0.077]***
	***Q96EY5***	***Cytoplasm; Nucleus***	***Nucleus[0.246]; Endosome[0.154]; Cytoskeleton[0.095]; Golgi apparatus[0.119];Plasma membrane[0.094]; Endoplasmic reticulum[0.075]***
	***P42858***	***Cytoplasm; Nucleus***	***Nucleus[0.285]; Cytoplasm[0.216]; Endoplasmic reticulum[0.071] Golgi apparatus[0.074]; Plasma membrane[0.096]***
	***Q9Y613***	***Cytoplasm; Cytoskeleton***	***Cytoskeleton[0.530]; Cytoplasm[0.146]; Nucleus[0.111]***

*Illustrations:*

[1] *True Subcellular Locations : denotes the labels from Hum-mPLoc 2.0 dataset (GOA database version 70.0 released March 10 2008)*;

[2] *Cytoskeleton[0.136]:the label NOT included in the original Hum-mPLoc 2.0 dataset, but validated TRUE by the latest Swiss-Prot database (* http://www.uniprot.org/uniprot/ *UniProt release 2011_11 Nov 16, 2011), where [0.136]* denotes the probability that the protein is assigned to the label *Cytoskeleton*;

[3] *Nucleus[0.115]*: *Non-target Label Hit* (wrong prediction), *NOT validated by the latest Swiss-Prot database (* http://www.uniprot.org/uniprot/ *UniProt release 2011_11 Nov 16, 2011), where [0.115]* denotes the probability that the protein is assigned to the label *Nucleus*;

As shown in Table 2 thru. Table 4, *MLMK-TLM* achieves 58.54%, 27.19% and 0 *LHR* (called *complete label hit rate CLHR*, *in bold font*) for *2*, *3* and *4* multiple subcellular locations (*optimistic* case), respectively (see Table 2); 56.87%, 25.58%% and 0 *LHR* (*CLHR*, *in bold font*) for *moderate* case (see Table 3); and 58.13%, 32.56% and 33.33% *LHR* (*CLHR*, *in bold font*) for *pessimistic* case (see Table 4). The results seem much more promising than 24.3% for 2-label hit rate, 3.6% for *3*-label hit rate and 6.7% for *4*-label hit rate, reported in the work [40]. The *complete label hit rate (CLHR)* for *pessimistic* case seems better than the *optimistic& moderate* case, because of the probability thresholds: 0.09 for *optimistic* case, 0.08 for *moderate* case and 0.07 for *pessimistic* case. Relax probability threshold would yields higher *Label Hit Rate* (*LHR*), but would yields higher *Non-target Label Hit Rate* (*NT*-*LHR*) at the same time. From Table 2 to Table 4, we can see that the *pessimistic* case shows higher *NT*-*LHR* than the *optimistic& moderate* case. The *complete label hit* means that all the true labels are correctly hit by the prediction, but it can not measure the model's misleading tendency, because the prediction is still likely to hit non-target labels. *Perfect Label Match Rate* (*PLMR*) is the perfect measure that demonstrates the model's multi-labelling ability with *zero misleading tendency*. As shown thru. Table 2 to Table 4, we can see from *PLMR* measure that the *optimistic* case is the best (42.92%, 13.95%, 33.33%), the *moderate* case the second (38.75%, 9.30%, 0) and the *pessimistic* case the third (34.58%, 9.30%, 0). We can see that even *MLMK-TLM*'s *Perfect Label Match Rate* is much better than the *Partial Label Match Rate* that was reported in the work [40]. Table 5 lists all the proteins of *perfect label match* in *optimistic*, *moderate* and *pessimistic* case, and the detailed probability outputs for the *perfect label match* proteins see Supporting Information (File S1 for *optimistic* case, File S2 for *moderate* case and File S3 for *pessimistic* case).

To further demonstrate *MLMK-TLM*'s multi-labelling ability, we list some proteins of *non-perfect label match* in Table 6 to show how the prediction varies from the true labels. Table 6 takes only 8 proteins for example and the full list of *non-perfect label match* proteins see Supporting Information (File N S1 for *optimistic* case, File N S2 for *moderate* case and File N S3 for *pessimistic* case). Take protein *O43663* in the *optimistic* case as an example, *O43663* is labelled *Cytoplasm* & *Nucleus* in the original *Hum-mPLoc* 2.0 dataset [5] (*GOA database version 70.0 released March 10 2008*), and the prediction not only hits the two true labels but also hit a non-target label *Cytoskeleton* with probability 0.136. From the latest *Swiss-Prot* database (*UniProt release 2011_11 Nov 16, 2011*, http://www.uniprot.org/), we can see that *Cytoskeleton* is truly assigned to protein *O43663*. The non-target labels validated as *TRUE Label* are underlined in Table 6. We can see that there are many underlined *TRUE Labels* in Table 6 for *optimistic*, *moderate* and *pessimistic* case. For example, *Cytoplasm* [0.166] & *Extracell* [0.103] for *P41222* in the *moderate* case; *Endoplasmic reticulum* [0.071], *Golgi apparatus* [0.074] & *Plasma membrane* [0.096] for *P42858* in the *pessimistic* case, etc., where the square bracketed number denotes probability. The underlined *TRUE Labels* demonstrates *MLMK-TLM*'s generalization ability rather than misleading tendency. Actually, *MLMK-TLM*'s misleading tendency is lower than the *NT-LHR* measures in Table 2 to Table 4 according to the latest *Swiss-Prot* database. No training proteins in *Hum-mPLoc* 2.0 dataset [5] are subjected to the subcellular localization pattern (*Nucleus*, *Cytoplasm*, *Endoplasmic reticulum*, *Golgi apparatus*, *Plasma membrane*) as *P42858*, whereas *MLMK-TLM* can correctly hit the five labels with different confidence levels, which is hard to achieve by the *nearest neighbour based* multi-label classifiers [19], [28], [29], [30], because the classifiers assigned to the query protein the labels that belong to the nearest training protein(s). *Hum-mPLoc 2.0* web server (http://www.csbio.sjtu.edu.cn/bioinf/hum-multi-2/) labels *O43663*, *P41222 and P42858* as follows: (1) *O43663: Nucleus*, without hitting *Cytoplasm* & *Cytoskeleton*; (2) *P41222: Endoplasmic reticulum*, *Golgi apparatus* and *Nucleus*, without hitting *Cytoplasm* & *Extracell*; (3) *P42858: Cytoplasm*, *Golgi apparatus* and *Nucleus*, without hitting *Endoplasmic reticulum* & *Plasma membrane*.

For both the *moderate* and the *pessimistic* case, the test proteins' own *GO* information is removed for the simulation of novel proteins, whereas *MLMK-TLM* can correctly predicts the test proteins' true labels and underlined *TRUE Labels* as illustrated in Table 2 to Table 6. The results show that *MLMK-TLM* has a good multi-labelling ability for novel *multiplex* human proteins. From Table 6, we also can see *MLMK-TLM* shows a certain misleading tendency, for example, *Nucleus* [0.115] & *Plasma membrane* [0.181] for *P30533* in the *optimistic* case; *Endoplasmic reticulum* [0.089] for *Q14145* in the *moderate* case; and *Endoplasmic reticulum* [0.315] for *Q9UHD9* in the *pessimistic* case, etc. *Hum-mPLoc 2.0* web server (http://www.csbio.sjtu.edu.cn/bioinf/hum-multi-2/) labels *P30533*, *Q14145 and Q9UHD9* as follows: (1) *P30533: Plasma membrane, Endoplasmic reticulum* and *Extracells*, hitting *non-target labels Plasma membrane* & *Extracells*; (2) *Q14145: Cytoplasm* and *Endoplasmic reticulum*, hitting *non-target label Endoplasmic reticulum*; (3) *Q9UHD9: Cytoplasm, Endoplasmic reticulum* and *Nucleus*, hitting *non-target label Endoplasmic reticulum*. Misleading tendency is an important factor that should be given attention for multi-label learning scenario. The advantage of probability outputs is to inform the biologists of the confidence level of each subcellular location, and thus help biologists make a rational decision.

## Discussion

In this paper, we propose a multi-label multi-kernel transfer learning model for human protein subcellular localization (*MLMK-TLM*), which o further extends our published work *GO*-*TLM* and *MK-TLM* to multi-label learning scenario, such that *MLMK-TLM* has the following advantages over the existing *GO*-based models [2], [4], [5], [14], [15], [16], [17], [18], [19], [20], [21], [22], [23], [24], [25], [26]: (1) proper homolog knowledge transfer with rational control over noise from divergent homologs; (2) comprehensive survey of model performance for novel protein; (3) multi-labelling capability with probability interpretation. As compared to single-label learning, multi-label learning is more complicated. In our work, we propose a multi-label confusion matrix and adapt one-against-all multi-class probabilistic outputs to multi-label learning scenario; meanwhile, we formally propose three multi-label learning performance measures: *LHR* (*Label Hit Rate*), *PLMR* (*Perfect Label Match Rate*) and *NT-LHR* (*Non-target Label Hit Rate*). *NT-LHR* is formally formulated to measure the model's misleading tendency. The experiments show that *MLMK-TLM* significantly outperforms the baseline model and demonstrates good multi-labelling ability for novel human proteins. Some findings (predictions) are validated by the latest *Swiss-Prot* database.

## Supporting Information

File S1 **Full list of** ***perfect label match*** **proteins in the** ***optimistic*** **case.** For each *multiplex* protein in the supplementary documents, there are three lines of description. The first line describes the protein accession; the second line describes the true label(s) of the proteins; and the third line gives the predicted label(s) of the protein. Each predicted label is followed by a squared bracketed number that denotes the probability the protein is predicted to the label.
(DOC)

File S2 **Full list of** ***perfect label match*** **proteins in the** ***modest*** **case.** The file format dittos.
(DOC)

File S3 **Full list of** ***perfect label match*** **proteins in the** ***pessimistic*** **case.** The file format dittos.
(DOC)

File N S1 **Full list of** ***non perfect label match*** **proteins in the** ***optimistic*** **case.** The file format dittos.
(DOC)

File N S2 **Full list of** ***non perfect label match*** **proteins in the** ***modest*** **case.** The file format dittos.
(DOC)

File N S3 **Full list of** ***non perfect label match*** **proteins in the** ***pessimistic*** **case.** The file format dittos.
(DOC)
