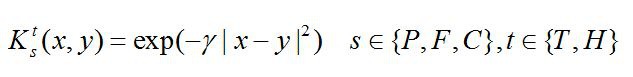# Correction: Multi-Label Multi-Kernel Transfer Learning for Human Protein Subcellular Localization

**DOI:** 10.1371/annotation/a4f3cb48-97ce-43e1-8bb4-eef6f8c389f8

**Published:** 2013-10-30

**Authors:** Suyu Mei

As a result of a problem in typesetting, there was an error in Figure 10. The correct version of the Figure is available here: