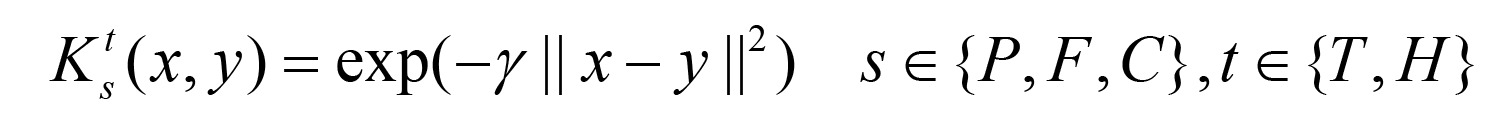# Correction: Multi-Label Multi-Kernel Transfer Learning for Human Protein Subcellular Localization

**DOI:** 10.1371/annotation/7e55b3cd-26bb-4d20-8112-bc2ed78be6dc

**Published:** 2013-12-13

**Authors:** Suyu Mei

There were errors in the correction posted on October 30, 2013. The correction is to Equation 10 rather than Figure 10, and the corrected formula contained an error. Please view the correct Equation 10 here: